# Myth or Truth: The Glass Forming Ability Class III Drugs Will Always Form Single-Phase Homogenous Amorphous Solid Dispersion Formulations

**DOI:** 10.3390/pharmaceutics11100529

**Published:** 2019-10-14

**Authors:** Piyush Panini, Massimiliano Rampazzo, Abhishek Singh, Filip Vanhoutte, Guy Van den Mooter

**Affiliations:** 1 Drug Delivery and Disposition, KU Leuven, Herestraat 49, 3000 Leuven, Belgium; piyush.panini@kuleuven.be (P.P.); massimiliano.rampazzo@kuleuven.be (M.R.); 2 Janssen Pharmaceutica, Turnhoutseweg 30, 2340 Beerse, Belgium; asing195@ITS.JNJ.com (A.S.); FVANHOUT@its.jnj.com (F.V.)

**Keywords:** amorphous solid dispersion, glass forming ability, GFA classification, glass transition, APIs, spray-drying, film-casting, mDSC, PXRD

## Abstract

The physical stability of amorphous solid dispersions (ASD) of active pharmaceutical ingredients (APIs) of high glass forming ability (GFA class III) is generally expected to be high among the scientific community. In this study, the ASD of ten-selected class III APIs with the two polymers, PVPVA 64 and HPMC-E5, have been prepared by spray-drying, film-casting, and their amorphicity at T0 was investigated by modulated differential scanning calorimetry and powder X-ray diffraction. It was witnessed that only five out of ten APIs form good quality amorphous solid dispersions with no phase separation and zero crystalline content, immediately after the preparation and drying process. Hence, it was further established that the classification of an API as GFA class III does not guarantee the formulation of single phase amorphous solid dispersions.

## 1. Introduction

The conversion of crystalline active pharmaceutical ingredients (API) to the amorphous state leads to the enhancement of their solubility and bioavailability [1]. This is because amorphous solids are thermodynamically in a high-energy state compared to their crystalline state. Therefore, the energy associated with the disruption of the lattice in the crystalline state is not required for solubilizing the APIs. However, beneficial improvement of dissolution kinetics of a pure API in its amorphous state is always at risk because of its capacity to convert into its more stable crystalline state during the storage or formulation process [2]. To address this poor stability of amorphous APIs, amorphous solid dispersion (ASD) has become a significant formulation strategy in recent years, whereby a drug is dispersed in a suitable and pharmaceutically acceptable polymer in the amorphous state [3,4]. The outcome provides greater stability of the drug in the amorphous state with improved dissolution characteristics compared to its crystalline state. Further, in the amorphous solid dispersion, molecular interactions between drug molecules and polymer may play an important role for their miscibility, which results in a single-phase homogenous solid system [5]. In contrast, limited or no miscibility between drug molecules and polymer may lead to the crystallization of the drug or the amorphous phase separation of the two components, resulting in poor physical stability. The practice of amorphous solid dispersion as a commercial formulation strategy has been amplified considerably, however, selecting an appropriate API and polymer for this purpose is still mainly based on trial and error [4,6]. Computational prediction and scientific understanding about the complex phenomenon of ASD formation, behavior, and its stability has been improved in recent years, but still needs further refinement [7]. It is often believed by the scientific community and pharmaceutical industry that API with a high glass forming ability can persistently form ASDs of high stability [8]. A study on ASDs of two structurally related drugs, Felodipine and Nifedipine, with different glass forming ability, revealed that Felodipine, having higher affinity to form a stable amorphous solid, was witnessed to form a more stable ASD with PVPVA [8]. It was observed that, despite having a similar structure, molecular mobility, and glass transition temperature, Nifedipine crystallizes more rapidly than Felodipine. The relative crystallization tendency of these APIs were reflected in their crystallization tendency from the ASDs.

The glass forming ability (GFA) of a material is defined by its ability to transform into its amorphous state [9]. Two approaches were recently reported, whereby the tendency of different APIs to become amorphous was investigated. All APIs were categorized into three classes, I, II, and III, based on their GFA. The first approach is based on the solidification of an API from its melt during a cooling cycle [9,10], while the other is based on the solidification tendency by rapid solvent evaporation from an API solution in an organic solvent or solvent mixture [11]. In case of the former classification system [9], it was proposed that when an API is crystallized during cooling (20 °C/min) from its melt, it belongs to “class I”. When crystallization is observed during reheating (10 °C/min), it is termed as “class II”, whereas in cases where no crystallization occurs during cooling/ heating, it is classified as “class III”. This classification system has been significantly improved in a recent study where a critical cooling rate (independent of the predefined cooling and heating rate) has been considered for the conversion of an API into its amorphous state [10]. In this case, a class I API can only be amorphized by a cooling rate larger than 750 °C/min. A class II API requires a cooling rate larger than 10 °C/min, whereas a class III API will have a critical cooling rate below 2 °C/min. Notably, the classification of an API into a particular GFA class may also be influenced by the method used to determine the GFA. For instance, Cinnarizine is classified as a class II API according to the approach based on the solidification of an API from its melt during a cooling cycle [9], whereas it is classified into class III according to the critical cooling rate approach [10].

Moreover, in the case of the classification system [11], based on rapid solvent evaporation using spin coating, the crystallization behavior was classified as rapid (for class I), intermediate (class II), and slow (class III). Class III API did not display crystallization behavior for seven-days. The expected limitation of this classification system would be the choice of solvent or solvent mixture, which can be used for the experiment. For example, Cinnarizine belongs to class II when dichloromethane (DCM) was used for sample preparation, whereas it fits into class III when a DCM–ethanol solvent mixture (1:1, *v*/*v*) was used for sample preparation. Further, Loratadine behaves as class III with DCM, but as class II if ethanol or 1:1 DCM–Ethanol (1:1, *v*/*v*) are selected as the solvent system [11].

Many of the class III APIs were recently successfully predicted in silico by statistical modeling using partial least-squares projection to latent structure–discriminant analysis (PLS-DA) [12], and are further based on molecular weight and their thermal properties [13]. Considering the outcomes from both ways of the classification system about the GFA of an API, it is established that class III API will exhibit a great tendency of transformation into its amorphous solid state. Recent exploration on the impact of the GFA class of APIs on the physical stability of their ASDs revealed that the ASD of class III API, on average, exhibit the highest physical stability compared to that of class II and class I APIs [14]. Henceforth, it is easy to misinterpret these results that class III API will always be able to produce homogeneous amorphous solid dispersions. The objective of the present study is to verify this speculation or assumption. 

For the current study, the APIs of high glass forming ability were selected and their amorphous solid dispersion was prepared by spray drying [15,16,17] and film-casting. The homogeneity (phase behavior) of the prepared ASDs was analyzed by modulated differential scanning calorimetry (mDSC) and powder X-Ray diffraction (PXRD) immediately after preparation (i.e., at *T* = 0). Their physical stability over a certain period was not performed. Every experimental condition for the preparation of ASDs and different instrumental parameters for their analysis were kept consistent. Differential scanning calorimetry is an important characterizing technique for the phase behavior of an ASD, wherein one glass transition (*T*_g_) event is considered to be an indication of a homogeneous and single phase ASD, and the presence of two glass transition events is interpreted as a sign of phase separation [18]. Further, the presence of a crystalline API can also be noticed by the appearance of the corresponding melting peak of the API. It is noteworthy that the presence of a single glass transition event on DSC thermograms may not always correspond to a single phase homogeneous ASD and it requires further characterization by complementary techniques, e.g., solid-state NMR [19,20]. However, for systems where the *T*_g_’s of API and polymer are far apart, the presence of a single *T*_g_ in their ASD formulations may be considered as a characteristic of a single-phase homogeneous amorphous solid. Furthermore, PXRD is also an important complimentary characterizing technique to study solid phases and the presence of crystalline API in the sample; the diffraction peak corresponding to the crystalline phase of an API may appear in the diffractogram.

All active pharmaceutical ingredients for the current study were selected based on the following criteria:

(i) They should belong to GFA class III in one of the two classification approaches, as described before, i.e., based on their crystallization either from the melt [9] or by the rapid solvent evaporation method [11].
(ii) They should dissolve in the same solvent. DCM was considered the first choice of solvent in this study due to its high volatility.

**Scheme 1:**
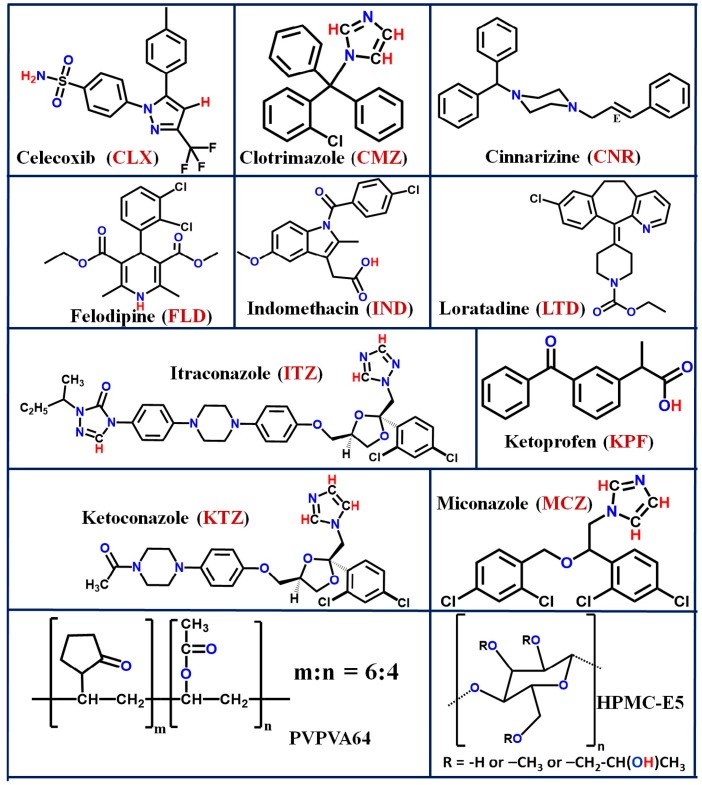
Chemical structure of APIs selected for the study (Table 1), along with the two polymers, PVPVA 64 and HPMC-E5. Hydrogen bond acceptor atoms are represented in blue, whereas hydrogen atoms, which can take part in the interactions, are in red.

A total of ten APIs were considered and are presented in Table 1. The diversities in the molecular structures of these API may also be expected to be important in this study as they have a different number of hydrogen bond donor and acceptor atoms (Scheme 1). 

Two polymers (Scheme 1) were selected in this study: (i) Poly(1-vinylpyrrolidone-*co*-vinyl acetate) (PVPVA 64) and (ii) Hydroxypropylmethylcellulose (HPMC-E5). The former is a 6:4 linear copolymer of *N*-vinylpyrrolidone and vinyl acetate. It has two potential proton acceptor functional groups (O=C as amide and ester group). Therefore, an API with a proton donor functional group can form a hydrogen bond with this polymer. HPMC-E5 has a strong donor of hydrogen bonds as –OH (hydroxyl) group along with the proton acceptor oxygen (from the methoxy groups) in the molecule. The –OH group in the polymer can be anticipated to interact with the hydrogen bond acceptor group in an API molecule. Therefore, the significance of hydrogen bonding between an API and polymer for phase behavior and glass stability of ASDs has also been investigated in this study.

## 2. Materials and Methods

Celecoxib, Clotrimazole, Felodipine, Ketoprofen, and Loratadine were purchased from TCI chemicals Europe N.V., Zwijndrecht, Belgium. Indomethacin and Ketoconazole were purchased from Alfa Aesar, Kandel, Germany and Acros Organics, Geel, Belgium, respectively. Cinnarizine and Miconazole were purchased from Fagron Belgium NV, Nazareth, Belgium and J&H Chemicals Co Ltd, Hangzhou, China, respectively. Itraconazole and two polymers, PVPVA 64 and HPMC-E5, were kindly supplied by Janssen Pharmaceutica, Beerse, Belgium. HPLC grade dichloromethane and methanol were purchased from Acros Organics, Geel, Belgium. All materials were used, as provided, in all experiments in this study and no further purification process was performed.

### 2.1. Preparation of Amorphous Solid Dispersions (ASDs) 

Two methods were considered to form ASDs of all APIs: Film casting and spray drying. Details of these experiments are described in the following sections. For both experiments, an API–polymer ratio 40:60 (*w*/*w*) was considered. 

For the film-casting experiments, only one polymer, i.e., PVPVA 64 was used with DCM as the solvent. The reason for this selection is that DCM is highly volatile, a useful property for the nature of this experiment, and both API and PVPVA 64 are soluble in this solvent.

Both PVPVA 64 and HPMC-E5 were considered in the spray drying experiments. PVPVA 64 concepts were spray dried with DCM, whereas HPMC-E5 concepts were prepared with 1:1 *w*/*w* DCM–methanol mixture. 

#### 2.1.1. Film-Casting

For the film-casting experiments, 100 mg of API was dissolved in 10 mL of DCM and stirred. 150 mg of PVPVA 64 was then added slowly to the solution with constant stirring until a clear solution was obtained. The clear solution was carefully and slowly cast over the Teflon surface (pasted on a glass plate) in two batches of equal amount. The glass plate was left in the hood for 24 h to completely dry the solvent. An opaque precipitate was observed in the case of CNR and ITZ, while films of other APIs were observed to be transparent on the Teflon surface. After 24 h, the solids were peeled from the surface, grinded with mortar and pestle, and kept in a vacuum oven at RT for 4 days. Subsequently, the materials were analyzed by modulated differential scanning calorimetry and powder X-ray diffraction.

#### 2.1.2. Spray Drying

API: Polymer (40:60 *w*/*w*) solutions were spray-dried using a Büchi Mini Spray Dryer B-191 equipped with a small cyclone. DCM was selected for preparation of ASDs with PVPVA 64, whereas 1:1 DCM-methanol was used for the ASDs with HPMC-E5. 400 mg of API and 600 mg of polymer were dissolved in 20 mL of solvent and stirred, as mentioned above. The spray dryer was preconditioned with respective solvent or solvent mixture for 5 min, setting the aspirator flow rate to 33 m^3^/h, the feed rate to 5 mL/min, and the atomizing airflow rate to 10 L/min. The inlet temperature was varied (70–80 °C) in order to keep the outlet temperature constant around 45 °C. After the experiment, the material was collected from the collector vessel and stored in a vacuum oven at RT for 4 days. Subsequently, samples were analyzed with PXRD and mDSC. 

### 2.2. Characterization of Materials 

#### 2.2.1. Powder X-Ray Diffraction (PXRD) 

All the samples were analyzed by PXRD on a PANalytical X’pert PRO powder diffractometer using an energy dispersive X’Cellerator detector (PANalytical, Almelo, The Netherlands). Measurement parameters: Cu Kα_1_ radiation (λ = 1.540598 Å); 45 kV; 40 mA; scanning interval 4° ≤ 2θ ≤ 40° at a step size of 0.0167° and 400 s counting time per step; and *T* = 25 °C in transmission geometry using Kapton^®^ polyimide foil sample holders. The X’pert Data Collector was used for data acquisition, and data analysis was performed using the X’Pert Data Viewer and X’Pert HighScore Plus (PANalytical, Almelo, The Netherlands).

#### 2.2.2. Modulated Differential Scanning Calorimetry (mDSC)

Differential scanning calorimetry measurements were performed using a Q2000 differential scanning calorimeter equipped with an RCS90 refrigerated cooling system (TA Instruments, Leatherhead, UK). Temperature calibration was performed for various heating rates (2, 5, and 10 °C/min) using *n*-octadecane, indium, and tin as a standard. The melting enthalpy calibration and validation were further performed using indium as a standard. A sapphire standard was used for heat capacity calibration using the modulating amplitude of 0.212 °C every 40 s with an underlying heating rate of 2 °C/min. Samples (weights range from 1–3 mg) were sealed in TA Instruments standard aluminum pans using TA Instruments standard aluminum lids. The sample chamber was continuously purged with inert dry N_2_ gas at a flow rate of 50 mL/min during the experiments. All experiments were performed in duplicate. Thermograms were analyzed using the Universal Analysis software package version 4.5A (TA Instruments). The melting point $T_{m}$ and the crystallization temperature $T_{x}$ were determined as the onset temperature of the heat flow curve. The glass transition temperature $T_{g}$ was determined as the inflection point temperature of the reversing heat flow curve. The enthalpy of fusion $\Delta H_{f}$ was determined as the integrated area of the melting endotherm in the total heat flow curve. The percentage (%) crystallinity of API was computed with respect to the heat of fusion of the pure API.

## 3. Result and Discussion

Overview of results after analysis by mDSC and PXRD of all ASD samples, prepared by film-casting and spray-drying is presented in Table 2.

Among all the APIs tested, only CLX and CNR belong to class II, based upon crystallization from the melt [9]. However, a recent study, based on time-temperature-transformation (TTT) diagram and critical cooling rate approach, classified CNR as class III [10] (Table 1). Furthermore, it is important to note that the assignment of the GFA class, based on fast crystallization from solvent or solvent mixture, might also depend on the choice of solvent or solvent mixture. For example, CNR is class II based on fast crystallization (by spin-coating) using DCM as solvent whereas it behaves as a class III drug when 1:1 (*w*/*w*) DCM and ethanol was considered as the solvent combination (Table 1). Further, LTD behaves as class III API when using DCM as the solvent, as opposed to the class II drug when 1:1 (*w*/*w*) DCM and ethanol is used. The remaining eight APIs considered in this study belong to class III based on crystallization from the amorphous state prepared using solvent based methods. 

**Figure 1:**
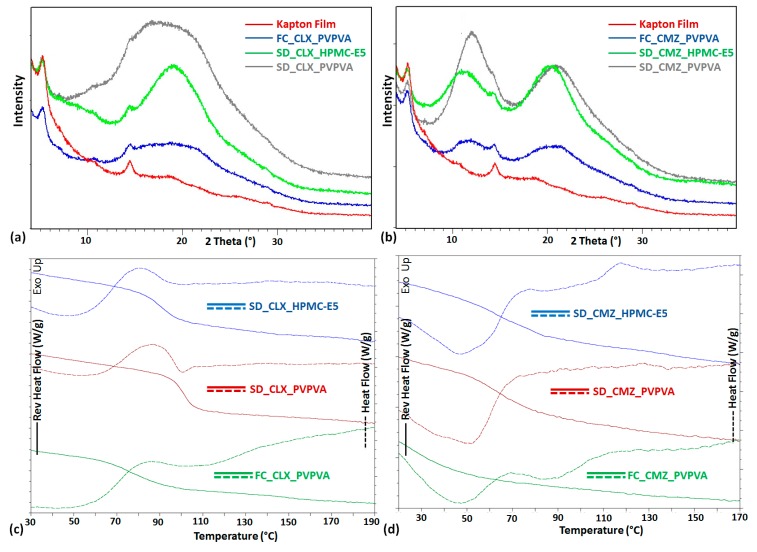
Powder X-Ray diffraction (PXRD) diffractogram of amorphous solid dispersions (ASD) sample, prepared by film casting and spray drying methods, with (**a**) Celecoxib and (**b**) Clotrimazole. (**c**,**d**) Their corresponding modulated differential scanning calorimetry (DSC) thermograms, showing reversing heat flow (as a solid line) and heat flow (as broken line) signals.

In the case of CLX and CMZ, ASDs prepared by both film casting and spray drying (API:Polymer = 40:60) experiments resulted in single-phase amorphous solids (Figure 1). No Bragg peaks were observed in the diffractogram upon analysis of these samples by powder X-Ray diffraction (Figure 1a,b). Further, mDSC thermograms of these samples displayed a single *T*_g_ (Figure 1a,b; Table 2). These measurements confirmed that CLX and CMZ ASDs were homogenous amorphous solids.

Therefore, for CLX and CMZ APIs, both PVPVA 64 and HPMC-E5 appeared to be suitable for the formation of the amorphous solid dispersion. For these APIs, film-casting experiments with PVPVA 64 also resulted in a single-phase amorphous solid. It was observed that the *T*_g_ of the spray dried ASDs displayed negative deviation of 10 to 24 °C from that obtained using the Gordon Taylor equation [23], with the exception for CLX-PVPVA ASD, wherein its experimental *T*_g_ was 11 °C higher than the calculated value by the Gordon–Taylor (GT) equation (Table 2). The GT equation predicts the *T*_g_ of a binary mixture where the components are completely miscible over the entire range of composition, assuming ideal mixing behavior, i.e., the Gibbs energy of mixing of the components is zero. The total magnitude of drug–polymer interactions in an API-polymer ideal mixture was equal to the total of API–API and polymer–polymer interactions in their components. The presence of a net specific interaction between the two components (strong H-bonds between drug and polymer) may lead to deviation of the experimental *T*_g_ value from the values calculated using the GT equation, and indicate the non-ideal mixing behavior between the components [18,24,25]. Furthermore, the presence of moisture or residual solvent as a plasticizer, preparation method, or condition [26,27], crystallization of drug molecules are other factors that can cause deviation in the glass transition temperature [18,28]. 

The above observations can also be explained in terms of potential API and polymer interaction in ASD. The presence of hydrogen bonding between API and polymer has a positive impact on the physical stability of the ASDs, as reported in a recent molecular simulation study on ibuprofen/PVPVA 64, ibuprofen/Eudragit, and fenofibrate/PVPVA 64 system [29]. In another study, it was observed that drug–polymer combinations capable of forming hydrogen bonding in the solution state leads to the formation of highly miscible amorphous solid dispersion and were more effective in preventing drug crystallization compared to the drug–polymer systems without such interactions [19].

Celecoxib consists of a strong H-bond donor such as the –NH_2_ group and strong nitrogen and oxygen acceptor atoms in the molecular structure (Scheme 1). Hence, a strong interaction of –NH_2_ with O=C as the amide and ester group in PVPVA 64 and methoxy groups in HPMC-E5 is expected. Furthermore, the –OH group in HPMC-E5 can also interact with the acceptor atoms (nitrogen and oxygen atoms) in Celecoxib. Therefore, a high value of *T*_g_ for CLX with PVPVA 64 (Table 2) can be explained by the presence of possible strong hydrogen bonding between the –NH_2_ group with O=C in the ASD. In the Celecoxib-PVP amorphous system, the presence of H-bonding between NH_2_ group of Celecoxib and C=O group of PVP was noticed by an FTIR spectroscopic study and computational simulation investigation of CLX and *N*-vinyl-2-pyrrolidone (a monomeric unit of PVP) [30]. Notably, a positive deviation of the glass transition temperature from that predicted by the GT equation was observed in the same study. The depression of *T*_g_ in film-casted CLX-PVPVA ASD, compared to those obtained by spray-drying, may be attributed to the fact that the former would have high a solvent content in the ASD.

The Imidazole group of CMZ can also form a moderately strong hydrogen bond with an acceptor atom via its three acidic H-atoms in the molecule [31] (Scheme 1). Consequently, ASDs of CMZ with PVPVA 64 and HPMC-E5 were formed with a single glass transition temperature (Table 2). The formation of a weak hydrogen bond by an acidic hydrogen atom in a molecule with strong acceptor atoms is very well established [32,33]. A very short C–H···O hydrogen bond, involving imidazolyl C–H with methanol and P=O group in cyclic phosphate, had been reported by Kumaraswamt and coworkers [34]. 

**Figure 2:**
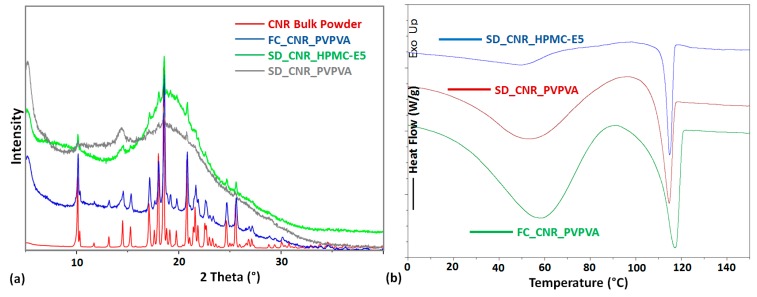
(**a**) Comparison PXRD patterns of bulk Cinnarizine with its ASD samples with PVPVA 64 and HPMC-E5. (**b**) Overlays of mDSC thermograms of ASDs of Cinnarizine, prepared by film casting and spray drying.

Figure 2 shows the PXRD and mDSC analysis of the ASD samples prepared with CNR by film casting and spray drying. PXRD diffractograms show the presence of diffraction peaks corresponding to CNR. Hence, it exhibits the presence of crystalline API in all samples prepared by film casting and spray drying. This was further confirmed by mDSC analysis. In the film-casted sample, the percentage of crystallinity was calculated to be 67%. Moreover, ASDs prepared by spray drying with PVPVA 64 and HPMC-E5 contained crystalline content up to 32% and 28%, respectively (Table 2). 

It is noteworthy that CNR does not possess strong H-bond donor atoms (Scheme 1). Its molecular structure is such that the two N-atoms in the molecule are not able to interact (phenomenon of steric hindrance) with any donor H-atom from a polymer. Hence, a possible interaction of CNR with PVPVA 64 or HPMC-E5 that requires stabilized ASD is unlikely. Hence, its crystallization from the ASD is not unexpected.

**Figure 3:**
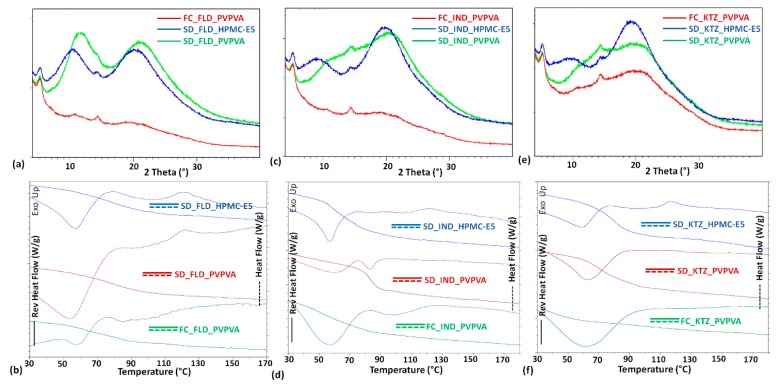
Overlay of PXRD patterns and mDSC thermograms of different ASDs samples of (**a**,**b**) Felodipine, (**c**,**d**) Indomethacin, and (**e**,**f**) Ketoconazole.

FLD, IND, and KTZ belong to class III based on both ways of classification, i.e., based on crystallization from the melt and rapid solidification from a solvent. The PXRD results confirm that their ASDs are amorphous solids. Further analysis with mDSC displays the presence of a single glass transition event and no sign of melting (Figure 3, Table 2). Hence, this proves that these are single-phase homogeneous amorphous solids with no crystallinity. It was observed that the *T*_g_ of spray dried ASDs of these APIs with PVPVA 64 was closer to the value calculated by the Gordon–Taylor equation (Table 2), indicating nearly ideal mixing behavior between API and polymer. However, for the ASDs prepared by film casting with PVPVA 64 and spray drying with HPMC-E5, the respective observed *T*_g_ value was significantly less than the calculated value. This may be due to the presence of a high level of residual solvents in these ASDs.

Apart from many H-bond acceptors, like nitrogen and oxygen atoms in these molecules, FLD had an –NH group and IND had a –COOH group as potentially strong H-bond donor atoms, whereas KTZ consisted of an imidazole group with three moderate H-bond donors (Scheme 1). In lieu of the above-mentioned donors and acceptors, a stable interaction with a polymer like PVPVA 64 and HPMC-E5 was anticipated and resulted in the formation of a single-phase homogeneous amorphous solid dispersion. The presence of O–H···O=C hydrogen bonding in IND-PVP/PVPVA system was confirmed previously by a spectroscopic study [35], and recently by solid state NMR studies [36]. MD simulations of FLD and HPMC system demonstrated the presence of a strong interaction between the two components in ASD [37]. Hydrogen bonding in the IND-PVP system was observed to be stronger than the FLD-PVP system according to IR studies on these systems [38]. This is expected as the –COOH group in IND is a stronger H-bond donor than the –N–H group in FLD. 

KTZ consisted of the weakest donors (as three hydrogens in the imidazole group) for H-bonding among these three APIs, and hence, the strength of H-bonding is also expected to be the weakest and was difficult to recognize or quantify by instrumental techniques. FTIR and ^13^C-NMR studies on KTZ-PVP K25 ASD confirmed that there was no strong and specific drug–polymer interactions in the solid dispersion [39]. It was described in the study that the stability of solid dispersion of KTZ in the polymer was due to the polymer anti-plasticizing effect.

**Figure 4:**
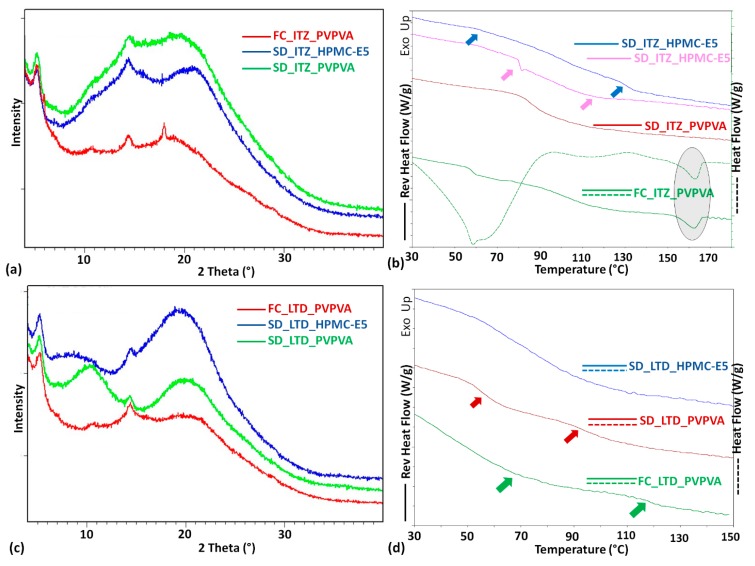
Overlay of PXRD patterns and mDSC thermograms of different ASDs samples of (**a**,**b**). Itraconazole and (**c**,**d**) Loratadine. Arrows in the mDSC thermograms mark more than *T*_g_ events (phase separation). The endothermic peak of melting event in the case of film-casted ITZ_PVPVA sample is marked by a shaded ellipsoid.

Furthermore, the PXRD diffractogram of a film-casted sample of ITZ with PVPVA 64 showed the existence of some crystallinity in the solid that was further supported by DSC analysis and the results showed the presence of up to 17% crystalline API in the sample (Figure 4a,b; Table 2). Conversely, ASDs of ITZ with PVPVA 64, prepared by spray-drying methods, were observed to display a single glass transition and no Bragg peaks in the corresponding PXRD diffractogram. The observed *T*_g_ was close to the calculated value by the Gordon–Taylor equation (Table 2), and hence was displayed closer to ideal mixing behavior of the components in the ASDs. Interestingly, ASDs of ITZ with HPMC-E5 displayed the formation of a phase-separated amorphous solid, as perceived by the analysis of their DSC thermogram (Figure 4b, marked with arrows; Table 2). 

ITZ had one triazole and one triazolone group in the molecule (Scheme 1), which consisted of moderately strong H-bond donor atoms [40,41]. These H-atoms can interact with the O=C groups (as amide and ester group) in PVPVA 64, promoting the formation of a single phase ASD. Further, the ITZ molecule had many potential H-bond acceptor atoms, which could interact with the –OH group in HPMC-E5 and formed a stable single phase ASD. However, this was not observed to be true in the current investigation with the experimental condition used in the study. The possible explanation would be the interaction with the moisture of the ITZ-HPMC-E5 amorphous solid dispersion during its formulation, as was reported recently [42]. It was observed that the presence of as little as 1% water in the solvent was enough to induce phase separation in the ITZ-HPMC-E5 spray dried solid. 

LTD is a class III API based on crystallization from its melt. However, its GFA class depended on the choice of solvent when “crystallization from solvent or solvent mixture” is considered. LTD behaved as a class III based on fast crystallization (by spin-coating) using DCM as solvent, whereas it displayed class II behavior when 1:1 DCM and ethanol or only ethanol was considered as a choice of solvent combination (Table 1). This property of LTD was not noticed in the formation of its ASDs. Its ASDs with PVPVA 64, either prepared by film-casting or spray-drying and using DCM as a solvent, displayed the formation of a phase-separated amorphous solid (Figure 4d**,** marked with arrows; Table 2). However, a single-phase amorphous solid dispersion of LTD was obtained with HPMC-E5, using 1:1 DCM and methanol as a solvent mixture for the spray drying (Figure 4). This phenomenon may be explained by the molecular structure of LTD (Scheme 1). It possessed two nitrogen and two oxygen atoms in the molecule, which interacted with the strong H-bond donor—OH group in the HPMC-E5. Hence, a single-phase amorphous solid dispersion with HPMC-E5 was obtained. In contrast, LTD did not have a potential H-bond donor atom, thus a strong interaction with PVPVA 64 was not feasible in its ASD, and consequently, a phase-separated amorphous solid was observed. However, a solid dispersion of 10% (*w*/*w*) LTD with PVP K30 was successfully obtained to enhance the bioavailability of the drug [43]. Therefore, it can be anticipated that LTD has very low miscibility in the polymer PVPVA 64 due to absence of or very low drug–polymer interaction. 

**Figure 5:**
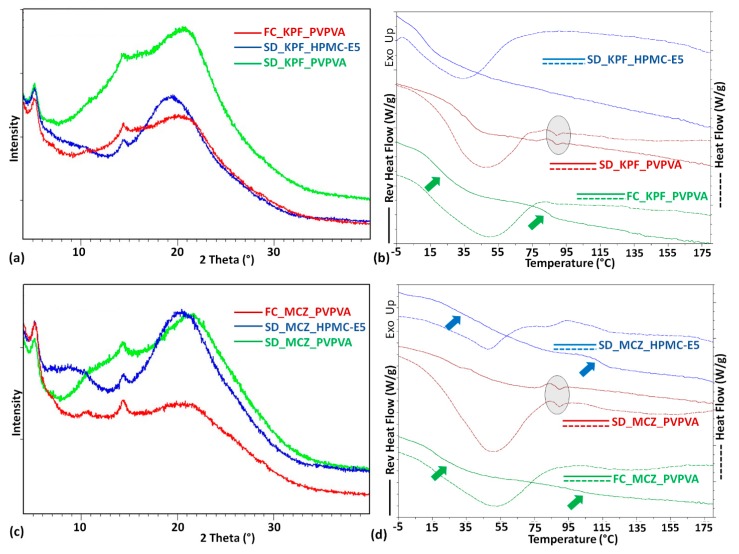
Overlay of PXRD patterns and mDSC thermograms of different ASDs samples of (**a**,**b**) Ketoprofen and (**c**,**d**) Miconazole. More than one *T*_g_ event is marked by arrows in the mDSC thermograms. The endothermic peak of melting events is marked by the shaded ellipsoid.

KPF and MCZ both belong to the GFA class III based on the two ways of classification (Table 1). Their film-casted samples with PVPVA 64 were phase-separated amorphous solids, whereas the presence of a very small crystalline (up to 1%) content was noticed in their ASDs with PVPVA 64, prepared by spray drying (Table 2, Figure 5). It was reported earlier that the presence of moisture might induce phase separation in MCZ-PVPVA amorphous solid dispersion [44]. It is noteworthy here that, since the API starts to crystalize immediately after preparation, an increase in the crystalline content will most likely take place with time in these solids. While KPF has a carboxyl (–COOH) group as a potential H-bond donor, it did not appear to form a stable interaction with PVPVA 64 (Scheme 1). A moderate H-bond donor imidazole group in MCZ was also not observed to be strong enough to form a stable interaction with PVPVA 64 in its ASD. 

ASDs of KPF, prepared by spray drying with HPMC-E5, resulted in the formation of a single-phase amorphous solid with a low *T*_g_ (less than 12 °C). This value was very low when compared to the value (72 °C) calculated by the Gordon–Taylor equation (Table 2). Hence, the miscibility of KPF with HPMC-E5 was far from the ideal behavior, which may have been due to the presence of a high amount of residual solvents or moisture in the ASD. This is why the stability of the ASDs of KPF with HPMC-E5 may be suspected. Moreover, a phase separated amorphous solid was obtained when MCZ was spray dried with HPMC-E5. Hence, it can be anticipated that KPF and MCZ do not form strong hydrogen bonds with the polymer HPMC-E5 that can guide the formation of a single-phase amorphous solid.

Solid dispersions of KPF with three hydrophilic polymers, namely, polyvinylpyrrrolidone (PVP) K30, PVPVA 64, and polyvinylalcohol (PVA) were prepared and characterized by Chan et al. with 30% drug loading [45]. It was observed in that study that the ASD of KPF with PVP K30 and PVPVA 64 resulted in a fully amorphous system, whereas a partially crystalline system was obtained with spray dried KTP PVA. The FTIR study confirmed strong drug–polymer interactions with PVP K30 and PVPVA 64 (both consist of C=O group with which –COOH from KPF can interact), while a weak drug–polymer interaction was observed in the solid dispersion of KPF with PVA (consist of hydrogen bond donor –OH group, similar as HPMC-E5). Therefore, the low *T*_g_ value for KPF-HPMC-E5 system, in the current study, may be related to a weak drug–polymer interaction and consequently low drug–polymer miscibility. Furthermore, at 40% drug loading, KPF-PVPVA ASD exhibited the beginning of drug crystallization in this study. This phenomenon may be attributed to the fact that the concentration of the drug in the polymer was higher than the solubility limit. This was also observed in case of the LTD-PVPVA system, wherein low (10%) drug loading reportedly gave miscible solid dispersion, while high drug loading (40% in the current study) resulted in a phase separated amorphous solid. 

Therefore, understanding the miscibility behavior of a drug in the polymer and the role of their mutual interaction are essential for the formulation of its amorphous solid dispersion, particularly in order to determine the maximal drug loading without the risk of drug precipitation or crystallization. A pristine class III API may be converted into an amorphous state easily, but the formulation of a successful and stable ASD will be governed by the nature of its miscibility or solubility behavior in the polymer matrix in the amorphous state. Amorphous drugs need to be mixed or dispersed molecularly into the polymer matrix so that drug crystallization or phase separation can be prevented. For a favorable mixing of the two components, their Gibbs energy of mixing, Δ*G*_mix_, should be negative in the following equation [22,24,28]:

Δ*G*_mix_ = Δ*H*_mix_ – *T*Δ*S*_mix_; (1)

where Δ*H*_mix_ and Δ*S*_mix_ are enthalpy and entropy of mixing at a constant temperature, *T*.

In the case of dispersion of small drug molecules in a polymer matrix, the entropy of mixing (Δ*S*_mix_) is positive, thus providing a favorable condition for their miscibility. However, Δ*S*_mix_ would be inversely related with the concentration of drug loading in its solid solution with a polymer. Hence, upon increasing the API loading in the polymer, at a particular concentration, an unfavorable situation for drug–polymer mixing may occur. This can lead to the phase separation or drug precipitation, particularly for a system where enthalpy of mixing is positive or less favorable. In addition, the enthalpy of mixing, Δ*H*_mix_, may have a major impact on drug–polymer miscibility behavior as its larger positive value may lead to amorphous–amorphous phase separation in solid dispersion. In a study by Rumondor et al., a favorable Δ*G*_mix_ was observed for FLD-polyvinylpyrrolidone (PVP) ASDs for as high as 70% API loading, compared to FLD-poly(acrylic acid) (PAA) and KTZ-PVP ASDs [46]. Based on IR and PXRD studies, the authors concluded that **s**trong drug–polymer interaction (hydrogen bonding) in FLD-PVP ASDs leads to favorable Δ*H*_mix_. In the case of KTZ-PVP ASDs, wherein KTZ interacts weakly with the polymer, a favorable Δ*G*_mix_ was observed only until 30% of API. Further, limited drug–polymer miscibility was detected in the case of FLD-PAA, which showed no drug–polymer interactions in the study. A favorable value of enthalpy of mixing can occur in a system where the total interaction between drug and polymer is stronger than the individual drug–drug and polymer–polymer interactions. For example, in a recent quantitative study using solid state ^13^C NMR on amorphous IND and IND-PVPVA systems, it was observed that by increasing the amount of PVPVA in IND-PVPVA solid dispersion formulations, the hydrogen bonded carboxylic acid dimers between two IND molecules are being consequently disrupted [36]. Phase separation in drug–polymer solid dispersions may lead to thermodynamically driven crystallization of the drug because of lower Gibbs energy of the crystalline phase compared to that of the amorphous phase. 

It has been well established that amorphous solid dispersions, prepared with drug loading below the saturation solubility of the drug in the polymer, will be thermodynamically stable and result in the formation of a single phase [14,47]. This will be independent of the GFA class of the drug. Therefore, in order to merely study the effect of the GFA class of API in the formation of single-phase homogeneous systems, it is very important to prepare the ASD, wherein drug loading is well above the maximum solubility limit. The maximum solubility of drug in the polymer is mostly observed to be less than 30%, when calculated by different analytical and computational methods [47,48]. For example, Knopp et al. recently studied drug–polymer solubility of CLX-PVPVA, IND–PVPVA, and FLD-PVPVA ASDs along with other drug–polymer systems by different methods [47]. It was observed that drug–polymer solubility at 25 °C for CLX-PVPVA, IND–VPVA and FLD-PVPVA are 26%, 22%, and 5% drug in the polymer, respectively, when calculated by the liquid analogue solubility approach, whereas those are 25%, 35%, and 6%, respectively, when calculated by melting point depression methods. Hence, in the current study with the drug loading of 40% in all ASD formulations, it can be expected that the concentration of the drug in the polymer is well above its maximum solubility limit. It is noteworthy that, in the current study, CLX-PVPVA, IND–PVPVA, and FLD-PVPVA were observed to exist as single phase ASDs. One of the explanations of this observation is most likely the phenomenon of kinetic trapping of APIs in the polymer matrix. It was established, in a previous study, that during the preparation of an ASD using spray drying, if solvent evaporation is fast enough to trap the API in the polymer matrix, then the API does not get enough time to crystallize and an ASD with drug loadings higher than its thermodynamically solubility limit, can be prepared [27]. Therefore, along with thermodynamic stabilization of ASDs, kinetics may play an important role in stabilization of APIs in the polymer matrix by minimizing its molecular mobility, thus preventing the crystallization of API or phase separation in ASDs.

## 4. Conclusions

In conclusion, it cannot be generalized that class III APIs, i.e., APIs with high glass forming ability will always produce acceptable amorphous solid dispersion. In the current study, out of 10 APIs, only CLX, CMZ, FLD, IND, and KTZ resulted in the formation of single-phase homogenous amorphous solids with no crystalline content by both methods for the preparation of ASDs: Film-casting with PVPVA 64 and spray drying with PVPVA 64/HPMC-E5. A possible explanation of these occurrences has been presented, based on the interaction between API and polymer, which can lead to their complete miscibility in the amorphous solid dispersion. There are many other factors such as miscibility of API in a polymer, choice of solvent/manufacturing method, and other kinetic factors that will play an important role in determining the physical stability landscape and surely warrants further systematic investigation. The role of different solvents or solvent mixtures in the stability of numerous ASDs of different APIs is going to be a prime focus of our future research.

## Figures and Tables

**Table 1:** List of active pharmaceutical ingredients (APIs) selected for the study.

Serial No.	API ^a^	Molecular Weight (g/mole)	GFA CLASS		Melting Point (°C)	Heat of Fusion (Δ*H*_f_, J/g)	Glass Transition Temperature (°C) [9]
			Based on Crystallization from Melt [9,10]	Based on Rapid Solvent Evaporation (Using Spin Coating) [11]			
1	Celecoxib (CLX)	381.37	Class II	Class III	160.5	93.5	58
2	Clotrimazole (CMZ)	344.84	Class III	Class III	141.5	88.5	30
3	Cinnarizine ^b^ (CNR)	368.51	Class III	Class II or III	119	110	7
4	Felodipine ^b^ (FLD)	384.26	Class III	Class III	142	72	45
5	Indomethacin ^b^ (IND)	357.79	Class III	Class III	159	110	45
6	Itraconazole ^b^ (ITZ)	705.64	Class III	Class III	166	84.5	58
7	Ketoconazole ^b^ (KTZ)	531.43	Class III	Class III	147	99	45
8	Ketoprofen ^b^ (KPF)	254.28	Class III	Class III	94	111.5	−3
9	Loratadine (LTD)	382.88	Class III	Class II or III	134	71.5	37
10	Miconazole (MCZ)	416.13	Class III	Class III	84	81.5	1

**^a^** These APIs were also tested by the slow evaporation method. Clotrimazole and Miconazole were partially amorphous, whereas Ketoprofen was mostly amorphous [11]; **^b^** These remained amorphous when stored at 20 °C above their glass transition temperature [21].

**Table 2:** Overview of experimental results.

ASD Sample ^a^	DSC Measurement			PXRD Pattern Signature
	Observed Glass-Transition Temperature, *T*_g_ (°C) ^b^	Calculated Glass-Transition Temperature, *T*_g_ (°C) ^c^ (Using Gordon Taylor Equation [22,23])	Melting Point (°C); Heat of Fusion, Δ*H*_f_ (J/g); % Crystallinity	
FC_CLX_PVPVA	75	89	-----	Amorphous solid
SD_CLX_PVPVA	100	89	-----	Amorphous solid
SD_CLX_HPMC-E5	90	114	-----	Amorphous solid
FC_CMZ_PVPVA	31	73	-----	Amorphous solid
SD_CMZ_PVPVA	63	73	-----	Amorphous solid
SD_CMZ_HPMC-E5	74	94	-----	Amorphous solid
FC_CNR_PVPVA	28	57	109.15; 29.56 67%	very high crystalline content
SD_CNR_PVPVA	44	57	109.15; 13.86 32%	Crystalline content
SD_CNR_HPMC-E5	51	81	112.31; 11.23 26%	Crystalline content
FC_FLD_PVPVA	63	82	-----	Amorphous solid
SD_FLD_PVPVA	76	82	-----	Amorphous solid
SD_FLD_HPMC-E5	78	106	-----	Amorphous solid
FC_IND_PVPVA	52	81	-----	Amorphous solid
SD_IND_PVPVA	83	81	-----	Amorphous solid
SD_IND_HPMC-E5	59	105	-----	Amorphous solid
FC_ITZ_PVPVA	59; 103	87	99.69; 1.722 153.83; 4.032 17%	Crystalline content
SD_ITZ_PVPVA	85	87	-----	Amorphous solid
SD_ITZ_HPMC-E5	83; 102	111	-----	Amorphous solid
FC_KTZ_PVPVA	30	82	-----	Amorphous solid
SD_KTZ_PVPVA	72	82	-----	Amorphous solid
SD_KTZ_HPMC-E5	78	105	-----	Amorphous solid
FC_KPF_PVPVA	16 85	53	-----	
SD_KPF_PVPVA	38	53	86.40; 0.2755 0.6%	Amorphous solid
SD_KPF_HPMC-E5	11	72	-----	Amorphous solid
FC_LTD_PVPVA	39; 118	76	-----	Amorphous solid
SD_LTD_PVPVA	54; 93	76	-----	Amorphous solid
SD_LTD_HPMC-E5	74	98	-----	Amorphous solid
FC_MCZ_PVPVA	16; 104	60	-----	Amorphous solid
SD_MCZ_PVPVA	39	60	86.29; 0.3815 1%	Amorphous solid
SD_MCZ_HPMC-E5	46; 114	82	-----	Amorphous solid

^a^ FC = Film-casted; SD = Spray-dried; e.g., SD_CLX_HPMC-E5 means the ASD sample was prepared by the spray drying method and consists of Celecoxib with HPMC-E5. ^b^ Average values are being reported. For some cases, more than one *T*_g_ was observed. ^c^ *T*_g_GT(*x*) = (*xT*_g_1 + *k*(1 − *x*)*T*_g_2))/(*x* + *k*(1 − *x*)); *T*_g_GT(*x*) is glass transition temperature of the mixture; *x* = *wt*. fraction of the component, *k* ≈ (*T*_g_1·*ρ*_1_)/(*T*_g_2·*ρ*_2_), *ρ* = density in amorphous state, “1” refers to the material with the lower *T*_g_, whereas “2” is for higher *T*_g_.

## List of Abbreviation

API	Active pharmaceutical ingredients
ASD	Amorphous solid dispersions
DCM	Dichloromethane
°C	Degree Celsius
FC	Film-Casting
FTIR	Fourier-transform infrared spectroscopy
GFA	Glass forming ability
HPMC-E5	Hydroxypropylmethylcellulose
mDSC	Modulated differential scanning calorimetry
PXRD	Powder X-Ray diffraction
PVPVA 64	Poly(1-vinylpyrrolidone-*co*-vinyl acetate)
SD	Spray Drying
*T*_g_	Glass transition temperature
Δ*H*_f_	Heat of Fusion
Δ*G*_mix_	Gibbs energy of mixing
Δ*H*_mix_	Enthalpy of mixing
Δ*S*_mix_	Entropy of mixing
